# Comparative Analyses of the Antiviral Activities of IgG and IgA Antibodies to Influenza A Virus M2 Protein

**DOI:** 10.3390/v12070780

**Published:** 2020-07-20

**Authors:** Kosuke Okuya, Nao Eguchi, Rashid Manzoor, Reiko Yoshida, Shinji Saito, Tadaki Suzuki, Michihito Sasaki, Takeshi Saito, Yurie Kida, Akina Mori-Kajihara, Hiroko Miyamoto, Osamu Ichii, Masahiro Kajihara, Hideaki Higashi, Ayato Takada

**Affiliations:** 1 Division of Global Epidemiology, Hokkaido University Research Center for Zoonosis Control, N20 W10, Kita-ku, Sapporo 001-0020, Japan; kokuya@czc.hokudai.ac.jp (K.O.); eguchi-na980@pref.miyagi.lg.jp (N.E.); manzoor@czc.hokudai.ac.jp (R.M.); ryoshida@czc.hokudai.ac.jp (R.Y.); t.saito@czc.hokudai.ac.jp (T.S.); yurie-kida@czc.hokudai.ac.jp (Y.K.); akinam@czc.hokudai.ac.jp (A.M.-K.); hirom@czc.hokudai.ac.jp (H.M.); kajihara@czc.hokudai.ac.jp (M.K.); 2 Influenza Virus Research Center, National Institute of Infectious Diseases, Gakuen 4-7-1, Musashimurayama, Tokyo 208-0011, Japan; ssaito@nih.go.jp; 3 Department of Pathology, National Institute of Infectious Diseases, Toyama 1-23-1, Shinjuku-ku, Tokyo 162-8640, Japan; tksuzuki@nih.go.jp; 4 Division of Molecular Pathobiology, Hokkaido University Research Center for Zoonosis Control, N20 W10, Kita-ku, Sapporo 001-0020, Japan; m-sasaki@czc.hokudai.ac.jp; 5 Laboratory of Anatomy, Department of Basic Veterinary Sciences, Faculty of Veterinary Medicine, Hokkaido University, N18 W9, Kita-ku, Sapporo 060-0818, Japan; ichi-o@vetmed.hokudai.ac.jp; 6 Division of Infection and Immunity, Hokkaido University Research Center for Zoonosis Control, N20 W10, Kita-ku, Sapporo 001-0020, Japan; hidea-hi@czc.hokudai.ac.jp; 7 Global Station for Zoonosis Control, Global Institution for Collaborative Research and Education (GI-CoRE), Hokkaido University, N20 W10, Kita-ku, Sapporo 001-0020, Japan

**Keywords:** influenza A virus, matrix 2 protein, antibody, IgA, budding inhibition, cross-protective immunity

## Abstract

The influenza A virus (IAV) matrix-2 (M2) protein is an antigenically conserved viral envelope protein that plays an important role in virus budding together with another envelope protein, hemagglutinin (HA). An M2-specific mouse monoclonal IgG antibody, rM2ss23, which binds to the ectodomain of the M2 protein, has been shown to be a non-neutralizing antibody, but inhibits plaque formation of IAV strains. In this study, we generated chimeric rM2ss23 (ch-rM2ss23) IgG and IgA antibodies with the same variable region and compared their antiviral activities. Using gel chromatography, ch-rM2ss23 IgA were divided into three antibody subsets: monomeric IgA (m-IgA), dimeric IgA (d-IgA), and trimeric and tetrameric IgA (t/q-IgA). We found that t/q-IgA had a significantly higher capacity to reduce the plaque size of IAVs than IgG and m-IgA, most likely due to the decreased number of progeny virus particles produced from infected cells. Interestingly, HA-M2 colocalization was remarkably reduced on the infected cell surface in the presence of ch-rM2ss23 antibodies. These results indicate that anti-M2 polymeric IgA restricts IAV budding more efficiently than IgG and suggest a role of anti-M2 IgA in cross-protective immunity to IAVs.

## 1. Introduction

Influenza A viruses (IAVs) have two glycoproteins, hemagglutinin (HA) and neuraminidase (NA), on the viral surface. IAVs are divided into subtypes based on the antigenicities of HA and NA, restricting the capacity of neutralizing antibodies to subtype-specific [1]. The matrix (M) gene of IAVs encodes two viral proteins, M1 and M2. The M1 protein is one of the most abundant structural components in IAV particles present on the inner leaflet of the viral envelope [2]. The M2 protein is a tetrameric integral membrane protein with an N-terminal extracellular domain (M2e) of 24 amino acids, a transmembrane domain of 19 amino acids, and a cytoplasmic tail of 54 amino acids [3]. The M2 protein possesses ion channel activity, which is important for virus entry [4]. Aside from the ion channel activity, the M2 protein possesses membrane scission activity, which is necessary to pinch off newly produced virus particles during the budding process [5]. Since previous studies have suggested that HA may initiate the budding event, and colocalization of M2 and HA on the infected cell membrane is normally observed in virus budding sites [5,6]. Although the M2 protein presents on the virus particle surface, its amount incorporated into each virion is low; approximately 16–67 M2 monomers for every 500 HA molecules [7,8]. However, since this protein is abundantly expressed on the virus-infected cell surface and its antigenicity is highly conserved irrespective of the IAV subtype, and the M2 protein is considered to be a promising target for antiviral drugs and universal IAV vaccines [9,10,11]. In fact, previous studies have demonstrated that the M2e-based immunization protected mice from lethal challenge with IAVs with various HA subtypes [12,13,14,15].

Secretory IgA (SIgA) antibodies are known to contribute to mucosal immunity against IAV infection [16,17]. IgA monomers are linked by a single small polypeptide, joining (J) chain, to form polymeric (dimeric, trimeric, and tetrameric) IgA antibodies [18]. SIgA antibodies are secreted to mucosal surfaces via transcytosis mediated by the polymeric immunoglobulin receptor (pIgR) expressed on the epithelial cell basolateral membrane. SIgA antibodies are released with the extracellular portion of pIgR (i.e., the secretory component (SC) [19].) Previous studies have shown that HA-specific polymeric SIgA antibodies neutralize IAVs more effectively than monomeric IgA and IgG antibodies [20,21,22]. Furthermore, we previously reported that anti-HA polymeric SIgA antibodies that did not inhibit cellular entry (i.e., non-neutralizing antibodies) reduced the release of IAV particles from infected cells, most likely due to tethering of virus particles on the cell surface [23]. These studies suggest that polymeric SIgA antibodies possess stronger antiviral activity than monomeric forms of antibodies. Indeed, intranasal immunization of mice with inactivated IAV particles, which induced polymeric SIgA, provided cross-protective immunity against various IAV strains, while subcutaneous immunization was only effective against the strain homologous to the immunogen [24,25].

In this study, we focused on the antiviral activities of M2e-specific antibodies. We previously produced an M2e-specific mouse monoclonal antibody (MAb), rM2ss23 IgG [26]. Although rM2ss23 IgG does not show neutralizing activity, it inhibits plaque formation of IAVs [26]. Similarly, M2e-specific MAb 14C2 (IgG), which is also a non-neutralizing antibody, is able to limit the growth of IAV in vitro [8]. These previous studies suggest that anti-M2 antibodies have the potential to inhibit virus budding from infected cells and may contribute to protective immunity against IAVs. We hypothesize that polymeric IgA antibodies might have a greater ability to restrict the virus budding process than IgG. Using the amino acid sequence of the rM2ss23 variable region, we constructed mouse-human chimeric rMss23 (ch-rM2ss23) IgA and IgG, which were assumed to recognize the same epitope, and compared their antiviral activities in vitro.

## 2. Materials and Methods

### 2.1. Cells and Viruses

Madin–Darby canine kidney (MDCK) cells [27] were maintained in Eagle’s minimum essential medium (MEM; Sigma-Aldrich, St. Louis, MO, USA) supplemented with 10% bovine serum (Gibco, Waltham, MA, USA), 100 U/mL penicillin, and 0.1 mg/mL streptomycin (Gibco, Waltham, MA, USA). Human embryonic kidney (HEK)-293T cells [28] were maintained in Dulbecco’s modified Eagle’s minimum essential medium (DMEM; Sigma-Aldrich, St. Louis, MO, USA) supplemented with 10% fetal bovine serum (Gibco, Waltham, MA, USA), 100 U/mL penicillin, and 0.1 mg/mL streptomycin (Gibco, Waltham, MA, USA). Expi 293F cells (Thermo Fisher Scientific, Waltham, MA, USA) were maintained in Expi293 Expression Medium (Thermo Fisher Scientific, Waltham, MA, USA) as described in the manufacturer’s instructions. After inoculation with IAVs, MDCK cells were cultured in MEM containing 0.3% bovine serum albumin (0.3% BSA/MEM) with 5 µg/mL trypsin (Gibco, Waltham, MA, USA). MDCK cells were maintained at 37 °C in 5% CO_2_. Expi 293F cells were maintained at 37 °C in 8% CO_2_ with an orbital shaker at 125 rounds per minute. IAV strains, A/Adachi/2/1957 (H2N2) (Adachi) and A/Aichi/2/1968 (H3N2) (Aichi), were propagated in MDCK cells and stored at −80 °C until use. Infectious titers were determined as plaque-forming units (PFU) with MDCK cells.

### 2.2. 5′-Rapid Amplification of cDNA Ends (5′-RACE)-PCR and Sequencing

Total RNA was extracted from the hybridoma cells producing rM2ss23 IgG [26] using an RNeasy Kit (Qiagen, Hilden, Germany) and reverse transcribed with a SMARTer RACE 5′/3′ Kit (Clontech, Shiga, Japan) using 5′ RACE CDS primer A (Clontech, Shiga, Japan). The heavy chain (VH) and light chain (VL) genes encoding the variable region were amplified by PCR using SeqAmp DNA polymerase (Takara, Shiga, Japan) as described in the manufacturer’s instructions. The amplified VH and VL genes were cloned into pCR-Blunt II-TOPO (Invitrogen, Carlsbad, CA, USA), and subjected to nucleotide sequencing using a version 3.1 BigDye Terminator Sequencing Kit (Applied Biosystems, Foster City, CA, USA) and Applied Biosystems 3130xl Genetic Analyzer (Applied Biosystems, Foster City, CA, USA). The primer sequences are listed in Table S1.

### 2.3. Expression and Purification of IgG and IgA Antibodies

Plasmids expressing recombinant rM2ss23 MAbs were constructed as reported previously [22,23]. Briefly, the VH and VL genes of rM2ss23 were amplified with restriction enzyme sites and ligated into α1H, γ1HC, and κLC vectors [22,29]. Expi 293F cells were cotransfected with γ1HC- and κLC-expressing plasmids using an Expifectamine 293 Transfection Kit (Gibco, Waltham, MA, USA) to express the human–mouse ch-rM2ss23 IgG antibody. For expressing ch-rM2ss23 IgA antibodies, Expi 293F cells were cotransfected with α1H-, κLC-, J chain-, and SC-expressing plasmids. ch-rM2ss23 IgG and IgA antibodies were purified from Expi 293F cell supernatants by using UNOsphere SUPrA (BioRad, Hercules, CA, USA) and CaptureSelect IgA (Invitrogen, Carlsbad, CA, USA), respectively. Each antibody preparation was concentrated using Amicon Ultra 30K (Merck Millipore, Darmstadt, Germany). Concentrated IgA antibodies were subjected to gel filtration chromatography (GFC) using a Superose 6 10/300 GL column (GE Healthcare, Little Chalfont, UK) with an AKTA avant 25 chromatography system (GE Healthcare, Little Chalfont, UK). Fractions were collected in phosphate-buffered saline (PBS) at a flow rate of 0.5 mL/min and analyzed by blue native polyacrylamide gel electrophoresis (BN-PAGE) using NativePAGE 4–16% Bis-Tris Protein Gels (Invitrogen, Carlsbad, CA, USA) with NativeMark (Invitrogen, Carlsbad, CA, USA), a molecular weight standard. Considering the results of BN-PAGE, the fractions were separated into three antibody subsets: monomeric IgA (m-IgA), dimeric IgA (d-IgA), and trimeric/tetrameric (or quadrimeric) IgA (t/q-IgA). Fractions for each IgA antibody subset were pooled and concentrated using Amicon Ultra 30K (Merck Millipore, Darmstadt, Germany). Negative control IgG and IgA antibodies (MAb B12) were generated as described previously [22,23]. Briefly, the DNA fragment encoding the VL and VH genes of the B12 clone was amplified by PCR using cDNA obtained from a single-sorted B cell from a healthy adult volunteer and MAb B12 IgG, m-IgA, and p-IgA were generated using the same method for ch-rM2ss23 antibodies. All antibodies were stored at −80 °C until use.

### 2.4. Expression of Recombinant M2

Recombinant M2 proteins as antigens for enzyme-linked immunosorbent assay (ELISA) were prepared as reported previously [23,30]. Briefly, HEK-293T cells transfected with the protein expression vector pCAGGS [31] encoding recombinant M2 genes derived from Adachi and Aichi using TransIT-LT1 (Mirus, Madison, WI, USA) were subjected to membrane protein extraction using a Eukaryotic Membrane Protein Extraction Reagent Kit (Thermo Fisher Scientific, Waltham, MA, USA). The extracted membrane proteins were diluted 1000-fold with PBS and used as antigens for ELISA. The PCR conditions including primer sequences are available in Table S1.

### 2.5. Enzyme-Linked Immunosorbent Assay (ELISA)

ELISA plates (Nunc Maxisorp, Invitrogen, Carlsbad, CA, USA) were coated with the prepared M2 antigens and blocked with 3% skim milk (Becton Dickinson, Franklin Lakes, NJ, USA) in PBS. ch-rM2ss23 IgG, m-IgA, d-IgA, and t/q-IgA antibodies diluted in 1% skim milk in PBS containing 0.05% Tween 20 (PBST) were plated in triplicate, and the bound antibodies were detected using horseradish peroxidase (HRP)-conjugated goat anti-human IgG (H + L) (109-001-003, Jackson Immuno Research, West Grove, PA, USA) or HRP-conjugated goat anti-human IgA (H + L) (ab97215, Abcam, Cambridge, UK). The reaction was visualized by adding 3,3′,5,5′-tetramethylbenzidine (TMB; Sigma-Aldrich, St. Louis, MO, USA) and absorbance at 450 nm was measured.

### 2.6. Surface Plasmon Resonance (SPR) Assay

The SPR assay was performed using Biacore 3000 (GE Healthcare, Little Chalfont, UK) as described previously [22,23]. Briefly, the synthetic Aichi M2e peptide with a C-terminal His-tag (Cosmo Bio, Tokyo, Japan) was immobilized on the surface of Sensor Chip NTA (GE Healthcare, Little Chalfont, UK) by using a NTA Reagent Kit (GE Healthcare, Little Chalfont, UK). After Aichi M2e immobilization (10 µg/mL for 180 s), the molecular interactions of M2e with ch-rM2ss23 IgG, m-IgA, d-IgA, and t/q-IgA (10 µg/mL) were analyzed with a contact time of 60 s and a dissociation time of 1050 s.

### 2.7. Neutralization Assay

Appropriately diluted IAVs (100 PFU) were incubated with serial dilutions of ch-rM2ss23 antibodies (0.01–100 µg/mL) and then inoculated into MDCK cells. Anti-HA MAb S139/1, which neutralizes both Adachi and Aichi [21,32], was used as a positive control antibody. After incubation with the virus-MAb mixture, the cells were washed with PBS and overlaid with 0.3% BSA/MEM containing 1.2% Avicel RC 591 (FMC BioPolymer, Philadelphia, PA, USA) [33] and 5 µg/mL trypsin. After 20-h incubation, the cells were fixed with methanol and blocked with 1% BSA in PBS. Plaques were stained with MAb S139/1, HRP-conjugated goat anti-mouse IgG (H + L) (115-035-062, Jackson Immuno Research, West Grove, PA, USA), and a 3,3′-diaminobenzidine substrate (Wako, Osaka, Japan).

### 2.8. Plaque Size Reduction Assay

MDCK cells seeded on 12-well plates (Corning, Corning, NY, USA) were infected with appropriately diluted IAVs (50–100 PFU) and overlaid with 0.3% BSA/MEM containing 5 µg/mL trypsin, 0.8% agarose S (Wako, Osaka, Japan), and IgG and IgA forms of ch-rM2ss23 or the control antibody (MAb B12). After incubation with or without MAbs for two days at 35 °C, plaques were visualized with immunostaining using the same methods as described above for the neutralizing assay. Plaque images on each well were scanned and the sizes of at least 30 plaques for each condition were measured using ImageJ analysis software (National Institutes of Health, Bethesda, MD, USA).

### 2.9. Sample Collection of Cell Lysates and Supernatants of Infected Cells

MDCK cells seeded on 12-well plates (Corning, Corning, NY, USA) were incubated with IAVs at a multiplicity of infection (m.o.i.) of 2.0 for one hour, and then washed with PBS. Subsequently, the infected cells were incubated in the presence or absence of the IgG and IgA forms of ch-rM2ss23 or MAb B12. After 8-h incubation, the supernatant was centrifuged and collected into new tubes. Laemmli sample buffer (BioRad, Hercules, CA, USA) containing 2-mercaptoethanol was added to the cells and lysates were collected for western blotting.

### 2.10. Western Blotting

Cell lysates and supernatants mixed with the sample buffer were incubated at 95 °C for 10 and 5 min, respectively. After 12% sodium dodecyl sulfate (SDS)-PAGE, separated proteins were transferred onto polyvinylidene fluoride (PVDF) membranes (Merck Millipore, Darmstadt, Germany). The PVDF membranes were soaked with 3% skim milk (Becton Dickinson, Franklin Lakes, NJ, USA) in PBS and washed with PBST. Each membrane was incubated with a mouse anti-M1 MAb (APH 6-23-1-6) [34] and a mouse anti-beta actin antibody (ab6276, Abcam, Cambridge, UK) as primary antibodies and subsequently with HRP-conjugated goat anti-mouse IgG (H + L) (115-035-062, Jackson Immuno Research, West Grove, PA, USA) as a secondary antibody. These antibodies were diluted with PBST containing 1.5% skim milk. The bound antibodies were visualized with Immobilon Western (Merck Millipore, Darmstadt, Germany). The amount of the viral M1 protein was semi-quantified based on the band intensity using an Amersham Imager 600 (GE Healthcare, Little Chalfont, UK).

### 2.11. Real-Time Reverse Transcription (RT)-PCR

Viral RNA was extracted from the cell supernatant using a QIAmp Viral RNA Mini Kit (Qiagen, Hilden, Germany) and subjected to real-time RT-PCR-based IAV gene detection using one-step SYBR prime script RT-PCR Kit II (Takara, Shiga, Japan). Primer sets specific for conserved regions of the IAV NP gene were used [23]. Quantification of the NP gene was performed using a standard curve generated by threshold cycle values obtained from 10-fold serial dilutions (covering 10^2^ to 10^6^ copies) of the A/Puerto Rico/8/1934 (H1N1) NP gene inserted into the pHH21 vector [35]. All samples were tested in triplicate. Average copy numbers of the viral genome in the supernatant of IAV-infected cells incubated without any MAb were set to 100%. Real-time RT-PCR conditions are available in Table S1.

### 2.12. Transmission Electron Microscopy (TEM)

TEM was performed as described previously [21,23]. Briefly, MDCK cells infected with Aichi at m.o.i. 1.0–2.0 were cultured with or without ch-rM2ss23 IgG, m-IgA, d-IgA, or t/q-IgA (1 µg/mL) for 8 h and fixed with 2.5% glutaraldehyde in 0.1 M cacodylate buffer (pH 7.4). The fixed cells were post-fixed with 2% osmium tetroxide and dehydrated with a series of ethanol gradients followed by propylene oxide, embedded in Epon 812 resin mixture (TAAB Laboratories Equipment Ltd., Berks, UK) and polymerized. Ultrathin sections (50 nm) were stained with uranyl acetate and lead citrate and examined with a JEM-1210 (JEOL, Tokyo, Japan) electron microscope at 80 kV.

### 2.13. Statistical Analysis

Data (band intensity, RNA copy number, and plaque size) were analyzed using GraphPad Prism version 8 for Windows (GraphPad Software, San Diego, CA, USA). One-way ANOVA followed by Tukey’s multiple comparison test was used to analyze each dataset as indicated in the figure legends. The following statistical values and symbols are used throughout the manuscript; * *p* < 0.05, ** *p* < 0.01, *** *p* < 0.001, **** *p* < 0.0001.

## 3. Results

### 3.1. Production of Mouse-Human Chimeric rM2ss23 IgG and IgA Antibodies

**Figure 1:**
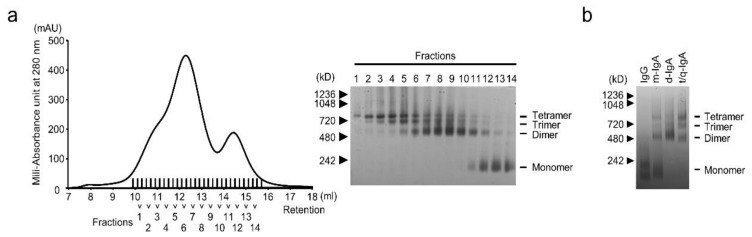
Purification of ch-rM2ss23 IgA. ch-rM2ss23 IgA antibodies were fractionated by gel filtration chromatography (GFC) with a Superose 6 10/300 GL column. A chromatogram demonstrating absorbance at 280 nm (shown in milli-absorbance units (mAU)) reveals two major peaks (**a**, left panel). Fractions covering the two peaks were subjected to blue native polyacrylamide gel electrophoresis (BN-PAGE) (**a**, right panel). Equal amounts (5 µg) of purified ch-rM2ss23 IgG, m-IgA, d-IgA, and t/q-IgA were used for BN-PAGE (**b**).

To compare the antiviral activities of the IgG and IgA anti-M2 antibodies, mouse-human chimeric IgG and IgA antibodies were generated based on the sequence of the rM2ss23 [26] variable region. The VH and VL genes of rM2ss23 were cloned into heavy and light chain expression plasmids for IgG and IgA and then Expi 293F cells were transfected with the constructed plasmids. To generate polymeric IgA antibodies, SC and J chain expression plasmids were cotransfected. After affinity purification of ch-rM2ss23 IgG and IgA from the supernatant, different forms of IgA antibodies were separated based on their molecular weights by GFC (Figure 1a); fractions 1–4, 6–10, and 12–14 were pooled for t/q-IgA, d-IgA, and m-IgA, respectively. The purity and molecular weights of the purified antibodies were validated (Figure 1b) and used for further experiments.

### 3.2. Binding Activities of ch-rM2ss23 IgG and IgA

**Figure 2:**
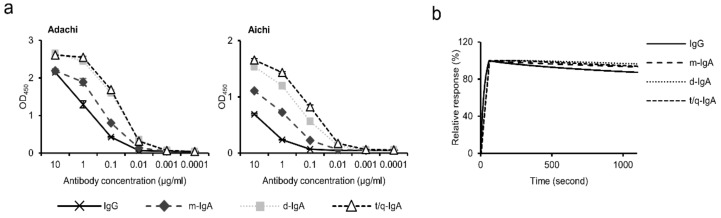
Comparison of binding to M2 antigens among the ch-rM2ss23 antibodies. Reactivities of IgG, m-IgA, d-IgA, and t/q-IgA (0.0001–10 µg/mL) to recombinant M2 proteins of A/Adachi/2/1957 (H2N2) (Adachi) and A/Aichi/2/1968 (H3N2) (Aichi) were measured in enzyme-linked immunosorbent assay (ELISA) (**a**). Binding dynamics of ch-rM2ss23 IgG, m-IgA, d-IgA, and t/q-IgA to the synthetic N-terminal extracellular domain of Aichi M2 peptide (**b**). Sensorgrams were adjusted (x = 0, y = 0: baseline, y = 100: binding) to allow comparisons between different antibody forms in terms of the dissociation rate.

We examined the binding activities of ch-rM2ss23 IgG and IgA antibodies using recombinant M2 proteins of Adachi and Aichi in ELISA. We found that the reactivities of polymeric IgA antibodies (i.e., d-IgA and t/q-IgA) to both M2 proteins tested were higher than that of m-IgA, and IgG showed lower OD values than any of the IgA forms (Figure 2a). However, since different secondary antibodies (HRP-labeled anti-IgG or anti-IgA antibodies) were used in ELISA, we assumed that it is not reasonable to directly compare the binding capacities between IgG and IgA antibodies in this assay. To further assess the binding activities of the antibodies, the binding dynamics of ch-rM2ss23 IgG, m-IgA, d-IgA, and t/q-IgA were investigated by SPR analysis to quantify the avidity of each ch-rM2ss23 to Aichi M2e (Figure 2b). We found that the SPR response of ch-rM2ss23 IgG decreased slightly faster than the IgA antibodies, indicating that the IgG only had a slightly higher dissociation rate (i.e., weaker binding) than IgA antibodies. Of note, there was no remarkable difference in dissociation rates among m-IgA, d-IgA, and t/q-IgA. These data suggest that the isotype conversion from IgG to the IgA1 backbone only had limited effects on the affinity/avidity of ch-rM2ss23. The increased OD values of d-IgA and t/q-IgA in ELISA might have been due to their polymeric forms giving multiple IgA monomers that provided more binding sites for the secondary antibody. Taken together, these data suggest that the antibody avidity of ch-rM2ss23 was minimally affected by IgA polymerization.

### 3.3. Reduction in Plaque Size in the Presence of ch-rM2ss23 IgG and IgA Antibodies

**Figure 3:**
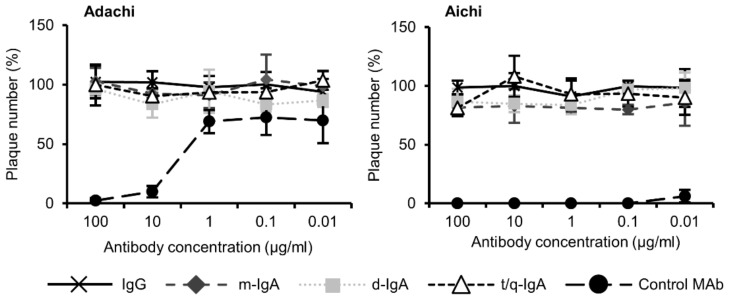
Neutralization tests of ch-rM2ss23 antibodies. Serial dilutions of ch-rM2ss23 IgG, m-IgA, d-IgA, and t/q-IgA and positive control neutralizing MAb (0.01–100 µg/mL) were mixed with each IAV strain, followed by plaque assays. Means and standard deviations of plaque numbers were calculated from three individual experiments. Relative plaque numbers to each control sample (i.e., cells incubated without MAbs) are shown.

We then confirmed that ch-rM2ss23 IgG and IgA antibodies did not possess neutralizing activity against either of two IAVs tested (Figure 3). These results were consistent with a study showing that the mouse rM2ss23 IgG antibody bound to Aichi M2, but did not show neutralizing activity [26].

**Figure 4:**
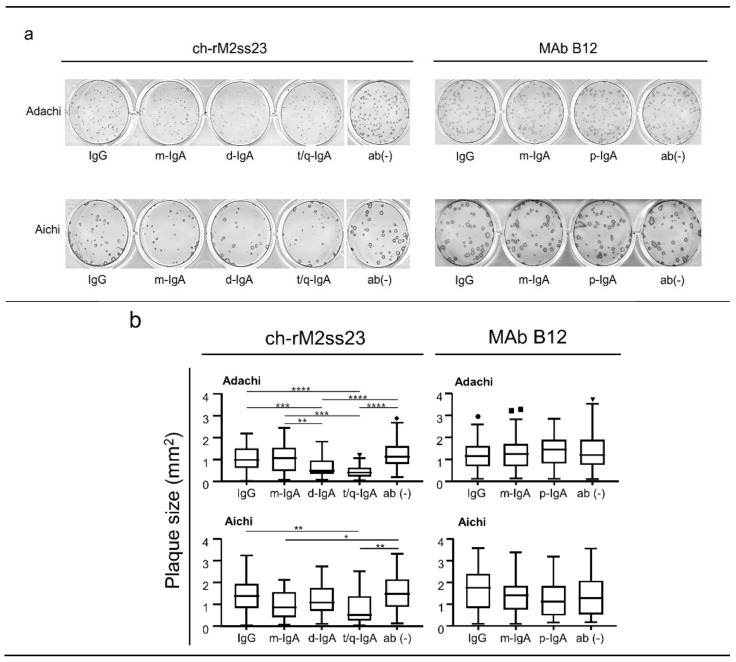
Reduced plaque sizes of IAVs in the presence of ch-rM2ss23 IgA antibodies. MDCK cells were infected with IAVs and incubated with or without MAb B12 and ch-rM2ss23 antibodies. Adachi- and Aichi-infected cells were incubated with MAbs at 10 µg/mL and 0.5 µg/mL, respectively. Plaques were stained as described in the Materials and Methods (**a**) and plaque sizes were measured for each well (**b**). Each box with a horizontal black line represents the interquartile range (IQR) and the median. The marks represent outlying plots located over 1.5× IQR from the upper quartile. Whiskers extend from the highest and lowest values within a fence. Asterisks indicate significant differences (* *p* < 0.05, ** *p* < 0.01, *** *p* < 0.001, **** *p* < 0.0001) determined using one-way ANOVA followed by Tukey’s multiple comparison test.

Previous studies have indicated that anti-M2 antibodies including rM2ss23, inhibit plaque formation of IAVs when the antibody is present in the overlay medium [8,26]. We therefore compared the ability to reduce plaque sizes among ch-rM2ss23 IgG, m-IgA, d-IgA, and t/q-IgA antibodies (Figure 4). MDCK cells infected with the IAVs were incubated with four different forms of ch-rM2ss23 and three different forms of the negative control MAb B12 (i.e., IgG, m-IgA, and polymeric [p]-IgA), and plaque sizes were measured. We found that the plaque sizes of Adachi and Aichi were significantly reduced in the presence of ch-rM2ss23 IgA antibodies, whereas IgG showed minimal reduction at these concentrations. The inhibitory effect of ch-rM2ss23 IgG was similar to that of the original mouse rM2ss23 [26]. Furthermore, ch-rM2ss23 t/q-IgA demonstrated a significantly higher ability to reduce the plaque sizes of Adachi than m-IgA. Plaque size reduction was not observed with the control MAb B12 antibodies. These results indicate that the ch-rM2ss23 t/q-IgA antibody possessed higher antiviral activity against Adachi and Aichi than the IgG or m-IgA antibody. We further analyzed the mechanism of the antiviral activity of ch-rM2ss23 t/q-IgA antibodies in the following experiments.

### 3.4. Reduction of Viral Particles Released from IAV-Infected Cells in the Presence of ch-rM2ss23 IgG and IgA Antibodies

**Figure 5:**
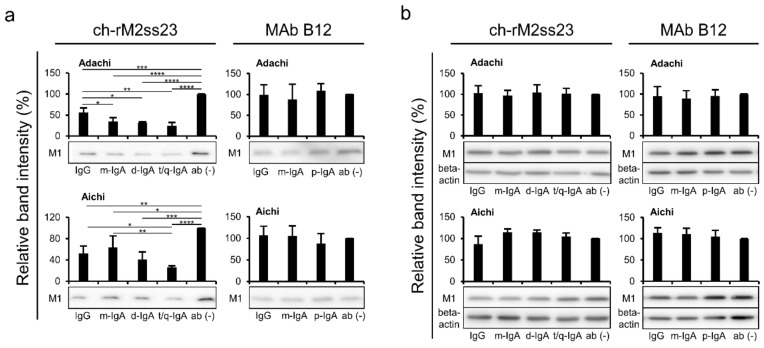
Detection of the viral protein in supernatants and lysates of IAV-infected cells. MDCK cells plated in 12-well plates were infected with IAVs at m.o.i. 2.0 and incubated with or without MAbs B12 and ch-rM2ss23 for 8 h at 35 °C. The cells infected with Adachi and Aichi were incubated with or without the MAbs at 10 µg/mL and 1 µg/mL, respectively. The M1 protein in supernatants (**a**) and cell lysates (**b**) was detected in western blotting. Beta-actin was also stained for cell lysate samples. Band intensities relative to each control sample (i.e., cells incubated without MAbs) are shown. Each experiment was performed three times and averages and standard deviations are shown. Asterisks indicate significant differences (* *p* < 0.05, ** *p* < 0.01, *** *p* < 0.001, **** *p* < 0.0001) found using one-way ANOVA followed by Tukey’s multiple comparison test.

Since non-neutralizing antibodies often have the potential to interfere with the virus release process [30,36], we examined the inhibitory effects of ch-rM2ss23 on virus release from MDCK cells infected with IAVs (Figure 5a). The amounts of M1 proteins were significantly lower in the supernatants of the ch-rM2ss23-treated cells than in those of the MAb-untreated cells. It was noted that t/q-IgA decreased the amount of M1 more significantly than IgG and/or m-IgA. There was no remarkable inhibitory effect with the control MAb B12 IgG, m-IgA, and p-IgA. Similar expression levels of the M1 protein of these two IAV strains were observed in cell lysates among the MAb treatments, indicating that viral protein synthesis was not affected by the treatment with the antibodies (Figure 5b).

**Figure 6:**
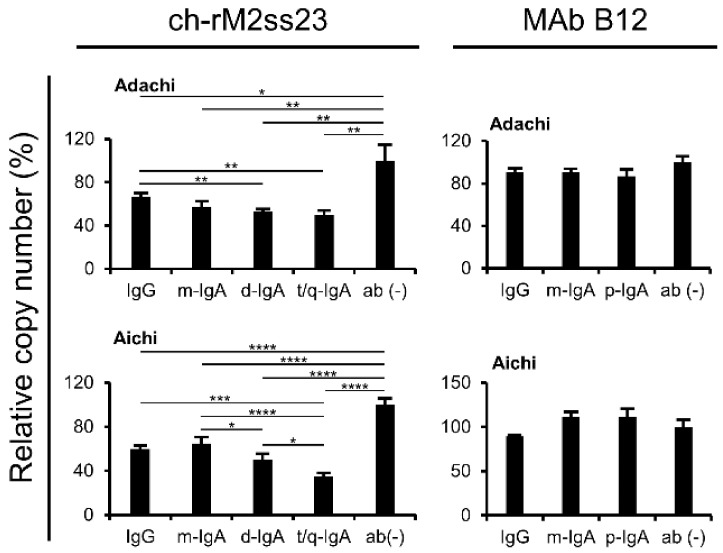
Detection of the viral RNA genome in the supernatants of IAV-infected cells. MDCK cells were infected with IAVs at m.o.i. 2.0 and incubated with or without MAb B12 and ch-rM2ss23 antibodies. The cells infected with Adachi and Aichi were incubated with or without the MAbs at 10 µg/mL and 1 µg/mL, respectively. The viral RNA genome was detected by real-time RT-PCR. The average copy number of the viral genome in the supernatant of IAV-infected cells incubated without MAbs was set to 100%. Each experiment was performed three times and the averages and standard deviations are shown. Asterisks indicate significant differences (* *p* < 0.05, ** *p* < 0.01, *** *p* < 0.001, **** *p* < 0.0001) determined using one-way ANOVA followed by Tukey’s multiple comparison test.

To further analyze the amounts of virus particles released from the IAV-infected cells, and viral RNA (NP gene) in the supernatants was quantified by real-time RT-PCR assays. We confirmed that the copy numbers of the NP gene of Adachi and Aichi were significantly decreased in the supernatants of the cells incubated with ch-rM2ss23 t/q-IgA compared to those of the cells incubated with IgG or m-IgA (Figure 6). Taken together, these results indicate that ch-rM2ss23 t/q-IgA had a higher antiviral activity than IgG and m-IgA to reduce the amounts of IAV particles released into cell culture supernatants.

**Figure 7:**
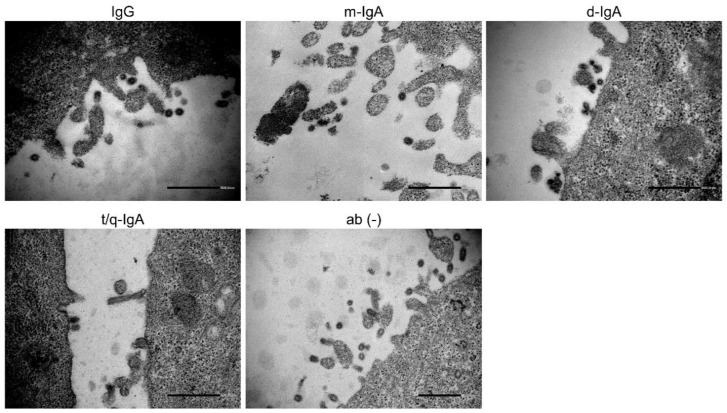
Electron microscopy of virus particles on the surface of Aichi-infected cells. MDCK cells were infected with Aichi at m.o.i. of 2.0 and incubated for eight hours with or without ch-rM2ss23 IgG, m-IgA, d-IgA, and t/q-IgA. Scale bars represent 500 nm.

Previous studies have reported that unusual aggregation and accumulation of virus particles were found on the IAV-infected cells cultured in the presence of MAbs, which inhibited the IAV release from infected cells [21,23]. To investigate whether ch-rM2ss23 antibodies aggregated virus particles on the surface of IAV-infected cells, Aichi-infected MDCK cells incubated with ch-rM2ss23 IgG, m-IgA, d-IgA, or t/q-IgA were observed by TEM. We found small numbers of virus particles in low proximity on the infected cells regardless of the presence of ch-rM2ss23 IgG and IgA antibodies as well as MAb-untreated cells (Figure 7), indicating that ch-rM2ss23 inhibited IAV release into the cell supernatant by a mechanism different from that of anti-HA MAbs reported previously [21,23].

## 4. Discussion

Intranasal immunization of mice induced cross-protective immunity against multiple IAV subtypes, most likely due to non-neutralizing SIgA antibodies [25,37]. Previous studies have shown that neutralizing antibodies are not the only indicator for protective immunity against IAV infection and that non-neutralizing antibodies, which are generally produced upon immunization and vaccination, also have important roles [30,38,39,40,41]. Compared to the IAV envelope glycoproteins (i.e., HA and NA), M2 protein is antigenically well conserved among all IAVs that caused past pandemics in humans, and it does not have a significant rate of antigenic drift [42,43]. Although the M2 protein works as a proton-selective channel and mediates the release of viral RNA into the cytosol [44,45], anti-M2 antibodies that inhibit virus entry into cells have rarely been reported. It is also known that the M2 protein is important for the budding of newly produced IAV particles from infected cells [7,46,47]. We therefore focused on the potential role of anti-M2 SIgA antibodies in cross-protective immunity against IAVs. In the present study, we compared the inhibitory effects of anti-M2 non-neutralizing MAbs (ch-rM2ss23 IgG, m-IgA, d-IgA, and t/q-IgA) on the virus budding and plaque formation processes of IAVs in vitro and found that ch-rM2ss23 t/q-IgA antibodies showed higher antiviral activity than IgG antibodies.

It has been shown that IgA antibodies often possess higher affinity to a single epitope than IgG due to altered flexibility of their constant heavy chains [48]. Accordingly, previous studies demonstrated that polymeric IgA antibodies showed higher antiviral activity than IgG [21,23,49]. In the present study, the dissociation rates of ch-rM2ss23 m-IgA, d-IgA, and t/q-IgA were slightly lower than that of IgG, which might contribute to the stronger budding inhibition of IgA antibodies (Figure 2b). Interestingly, however, although there was no remarkable difference in the antibody avidity among ch-rM2ss23 m-IgA, d-IgA, and t/q-IgA, t/q-IgA had relatively higher antiviral activity. This is most likely due to the multiplicity of antigen binding sites in a single t/q-IgA molecule.

Our previous studies found aggregation and accumulation of virus particles on the IAV-infected cells cultured in the presence of anti-HA MAbs that inhibited the IAV release from the cells [21,23]. Since the M2 protein is abundantly expressed on the surface of IAV-infected cells as well as the HA protein [8], we assumed that ch-rM2ss23 antibodies likely bonded to the M2 protein on the cell surface and tethered progeny virus particles newly produced from infected cells, as was shown with the anti-HA antibodies [21,23]. However, virus particles were not accumulated or agglutinated in the presence of ch-rM2ss23 IgG and IgA antibodies (Figure 7), indicating that ch-rM2ss23 inhibited IAV release into cell supernatants via a mechanism different from that of the anti-HA MAbs reported previously [21,23]. In the normal architecture of viral budding sites, viral integral membrane proteins (i.e., HA, NA, and M2), NP, and M1 proteins interact with each other [50]. Previous reports suggest that colocalization of HA and M2 is necessary for IAVs to effectively egress from the cells [5,37,44]. Thus, it might be possible that the distribution of M2 proteins on the infected cells was altered by ch-rM2ss23 IgA antibodies and the disrupted M2 localization interfered with the interaction with HA, leading to the reduction of newly produced virus particles from the infected cells.

Polymeric IgA antibodies are transferred intracellularly via pIgR to the apical membrane of epithelial cells and some of those antibodies are known to inhibit viral protein functions during transcytosis [51,52,53]. Intriguingly, previous studies demonstrated that polymeric IgA antibodies effectively inhibited rotavirus and measles virus replication via this mechanism (i.e., intracellular neutralization) [51,52]. Furthermore, it was also shown that anti-HA IgA, but not IgG, interacted with HA proteins newly synthesized in the IAV-infected cells, and subsequently reduced the viral growth [54]. Anti-M2 IgA antibodies, therefore, may have the potential to bind to M2 molecules in the IAV-infected cell and readily interfere with the function of M2 intracellularly, which may result in inhibiting virus particle formation. Further studies are needed to confirm whether anti-M2 IgA antibodies contribute to intracellular neutralization.

In conclusion, our data suggest that M2-specific polymeric IgA antibodies exhibit enhanced antiviral activity compared to IgG. Our findings support the idea that intranasal vaccination may provide cross-protective immunity to IAVs by inducing polymeric SIgA antibodies including anti-M2 IgA, that inhibit the budding process of progeny virus particles from infected cells. Further studies are needed to confirm the contribution of M2-specific SIgA antibodies to protection from IAV infection in vivo.

## Supplementary Materials

The following are available online at https://www.mdpi.com/1999-4915/12/7/780/s1. Table S1. Primer sequences and PCR condition.
