# Supplementary material for: Comparative Analyses of the Antiviral Activities of IgG and IgA Antibodies to Influenza A Virus M2 Protein
Source: Viruses. 2020 Jul 20;12(7):780. doi: 10.3390/v12070780 (PMC7411592; doi:10.3390/v12070780)
Supplement: Supplementary file 1 [file viruses-12-00780-s001.pdf]

viruses

MDPI

Supplementary Materials: Comparative Analyses of the Antiviral Activities of IgG and IgA Antibodies to Influenza A Virus M2 Protein

Table S1. Primer sequences and PCR condition.

**Primers Sequences for 5′RACE-PCR and Sequencing**	
mIgG1 H const-R	ATAGACAGATGGGGGTGTCGTTTTGGC
Mouse-IgK (3'const)	GGATACAGTTGGTGCAGCATC
Age1-IgG1 HC-F	GCAACCGGTGTACATTCCGAGGTGAAGCTGGTG
Sal1-IgG1 HC-R	TGGTCGACGCTGAGGAGACGGTGAC
Nhel-IgA HC-R	GGGCTAGCTGAGGAGACGGTGACTGA
Age1-Ig kappa LC-F	CAACCGGTGTACATTCAGATGTTGTGATGACC
BsiW1-Ig kappa LC-R	ACCGTACGTTTCAGCTCCAGCTTGGTC
M13F	GTAAAACGACGGCCAG
M13R	CAGGAAACAGCTATGAC
**Primers Sequences for Recombinant M2**	
Adachi-M2-F (ClaI)	TACGATCGATATGAGTCTTTTAACCGAGG
Aichi-M2-F (ClaI)	TACGATCGATATGAGCCTTCTAACCGAGG
Infl-A_M2-R (SphI)	GTAGCATGCCTATTACTCCAGCTCTATGC
**PCR Condition (KOD One; Takara)**	
Denaturation	98 °C 10 s
Annealing	50 °C 5 s
Extension	68 °C 20 s
30 cycles	
**Primers Sequences for Real-Time RT-PCR**	
NP 972F	CAAGAGTCAGCTGGTGTGGA
NP 1160R	GCCCAGTACCTGCTTCTCAG
**Real-time RT-PCR Condition (One-Step SYBR Prime Script RT-PCR kit II; Takara)**	
Hold stage	42 °C 5 min, 95 °C 10 s
PCR stage	95 °C 5 sec, 60 °C 32 s (40 cycles)
Melting curve stage	95 °C 15 s, 60 °C 1 min, 95 °C 15 s
